# Comparison of olive leaf, olive oil, palm oil, and omega-3 oil in acute kidney injury induced by sepsis in rats

**DOI:** 10.7717/peerj.7219

**Published:** 2019-07-09

**Authors:** Maria Fátima de Paula Ramos, Olvania Basso Oliveira, Alceni do Carmo Morais Monteiro de Barros, Clara Versolato Razvickas, Edson de Andrade Pessoa, Rinaldo Florêncio da Silva, Ana Maria Soares Pereira, Marcia Bastos Convento, Fernanda Teixeira Borges, Nestor Schor

**Affiliations:** 1 Nephrology Division, Department of Medicine, Universidade Federal de São Paulo, São Paulo, SP, Brazil; 2 Morphology and Genetics Division, Universidade Federal de São Paulo, São Paulo, SP, Brazil; 3 Biotechnology, Universidade de Ribeirão Preto, Ribeirão Preto, SP, Brazil; 4 Interdisciplinary Postgraduate Program in Health Sciences, Universidade Cruzeiro do Sul, São Paulo, SP, Brazil

**Keywords:** Lipopolysaccharide, Olive oil, Omega-3 oil, Palm oil, Kidney function, Inflammation

## Abstract

**Background:**

Hypotension, increased production of reactive oxygen species, and inflammation are all observed in experimental models of sepsis induced by lipopolysaccharide (LPS).

**Purpose:**

The aim of this study was to evaluate the effects of an ethanolic extract of Brazilian olive leaf (Ex), Brazilian olive oil (Olv), Ex + Olv (ExOlv), and palm oil (Pal) in comparison to the effects of omega-3 fish oil (Omg) in a rat model of sepsis-induced acute kidney injury.

**Materials:**

Wistar rats were divided into seven groups (seven per group), which were either untreated (control) or treated with LPS, LPS + Ex, LPS + ExOlv, LPS + Olv, LPS + Omg, or LPS + Pal.

**Results:**

Lower values of creatinine clearance and blood pressure were observed in the LPS-treated group, and these values were not affected by Ex, Olv, ExOlv, Pal, or Omg treatment. Mortality rates were significantly lower in rats exposed to LPS when they were also treated with Ex, ExOlv, Olv, Pal, or Omg. These treatments also decreased oxidative stress and inflammation (Tumor necrosis factor alpha, interleukin-1 beta) and increased interleukin-10 levels and cell proliferation, which were associated with decreased apoptosis in kidney tissue.

**Conclusion:**

Ex and Pal treatments were beneficial in septic rats, since they increased survival rate and did not aggravate inflammation. However, the most effective treatments for septic rats were Olv in comparison to Omg. These natural food substances could enable the development of effective therapeutic interventions to sepsis.

## Introduction

Acute kidney injury (AKI) is a syndrome characterized by an acute loss of renal function and is associated with an increased mortality rate (over 50%) and the development of chronic kidney disease (Liano & Pascual, 1996; Waikar, Liu & Chertow, 2008). AKI is a newly classified disease, which replaced the concept of traditional acute renal failure first described in 1952 (Smith, 1952) and was proposed in order to improve clinical diagnosis of the disease (Makris & Spanou, 2016).

Acute kidney injury is also very common in the setting of sepsis (Makris & Spanou, 2016). Our knowledge of AKI comes mainly from animal studies, where ischemia-reperfusion, toxic injury, and septic models are widely studied (Singh et al., 2012). Considering the complexities of sepsis, investigators rely heavily on in vivo models, the vast majority of which are based on rodents (Deitch, 1998).

Toxemia models often are used to study sepsis and its associated pathological mechanisms. Several injectable stimulatory agents have been used, including lipopolysaccharide (LPS) from the outer membrane of gram-negative bacteria, CpG DNA, synthetic lipopeptides, and zymosan. However, a single injection of endotoxin, or LPS, is the most commonly used toxemia model (Buras, Holzmann & Sitkovsky, 2005; Deitch, 1998).

The pathophysiology of septic AKI is complex. In 1992, the term systemic inflammatory response syndrome was developed, including a definition of sepsis as the presence of this systemic inflammatory response as a result of infection (Bone et al., 1992; Levy et al., 2003; Taniguchi et al., 2017). Recently proposed changes to this definition reflect the heterogeneous nature of this syndrome (Singer et al., 2016), where endothelial dysfunction, inflammation via the secretion of cytokines, apoptosis, and oxidative stress have all been implicated in the pathogenesis of sepsis (Mikacenic et al., 2017).

Pertinent to this, sepsis is characterized by excessive production of both reactive nitrogen species and reactive oxygen species (ROS) in affected organs due to mitochondrial dysfunctional and modified anti-oxidative status, and in the circulation due to activated immune system cells and endothelial cells (Andrades et al., 2011; Dare et al., 2009; Berger & Chioléro, 2007).

In recent years, there has been much research into effective therapies for the prevention and treatment of AKI. In view of this, we aimed to evaluate the potential of natural Brazilian products in a rat model of sepsis-induced AKI.

The olive tree (*Olea europaea* L., Oleaceae) is found in many different parts of the world, including Brazil. Olive leaves have been shown to have anti-atherosclerotic, hypotensive, antioxidant, anti-inflammatory, and hypocholesterolemic effects (Lockyer et al., 2017; Casas et al., 2016; Castrogiovanni et al., 2016). Olive oil is more potent than fish oil at reducing septic pulmonary dysfunction in rats, by enhancing the antioxidant defense system and suppressing oxidative stress (Glatzle et al., 2007).

Oil palm natural (*Elaeis guineensis* *Jacquin*) produces two different kinds of oil namely, palm oil (Pal) and palm kernel oil. Pal is extracted from the fleshy mesocarp (outer pulp). It is rich in vitamin E, which inhibits cholesterol synthesis and protects the cell membrane from lipid peroxidation, thus conferring a protective effect against cardiovascular and neurodegenerative diseases and supporting the treatment of cancer (Mancini et al., 2015). The high vitamin E content in Pal may help to counterbalance proinflammatory effects. Pal has been shown to have anti-inflammatory effects in myocardial tissue in LPS-induced sepsis models (Katengua-Thamahane et al., 2014).

Fish oil contains a high concentration of omega-3 polyunsaturated fatty acids. Omega-3 fatty acids and their longer-chain downstream products, such as eicosapentaenoic acid (EPA), docosapentaenoic acid, and docosahexaenoic acid (DHA) have been reported to be beneficial in models of critical illness (Firat et al., 2017). The effects of omega-3 fatty acids include modulation of the immune response, inhibition of proinflammatory factors, and promotion of anti-inflammatory mediators. Omega-3 fatty acids have also been shown to be beneficial in models of chronic renal failure (Mas et al., 2016), and in sepsis in humans (Lu et al., 2017; Chen et al., 2018; Svahn et al., 2016) and animals (Dehkordi et al., 2015; Frimmel et al., 2016).

Therefore, the present study aimed to compare the effects of an extract of Brazilian olive leaf (Ex), Brazilian olive oil (Olv), Pal, and omega-3 fish oil (Omg) in an experimental rat model of sepsis-induced AKI.

## Materials and Methods

### Induction of sepsis

Rats were intraperitoneally injected with 10 mg/kg *Escherichia coli* LPS (serotype 055: B5; Sigma, St Louis, MO, USA), dissolved to 0.9% in distilled water. The control (CTL) group received the same volume of distilled water only. To begin the experimental protocol, sepsis was stimulated by LPS treatment after 24 and 48 h. Rats were subsequently untreated or treated with test substances after 24 and 48 h. The treatment protocols are described in detail below.

### Treatment protocol: Brazilian olive

The oil and fresh leaves of Brazilian olive trees were provided by Cerro dos Olivais, located in Picada Grande, Caçapava do Sul-RGS, Brazil, associated with the Associação Rio-Grandense de Olivicultores. A lyophilized extract was prepared from the olive leaves by Universidade de Ribeirão Preto (UNAERP/SP) using ethanol as the solvent.

Ethanol has been reported to be the most suitable solvent for preparing *O. europaea* L. (olive leaf extracts) with a high content of phenolic compounds (Lafka et al., 2013; Pérez-Bonilla et al., 2013).

Olive leaf extracts were analyzed at Phytobios/Centroflora Brazil, using a high-performance liquid chromatography system (Merck-Hitachi LaChrom Elite; Hitachi, Tokyo, Japan), equipped with a UV/vis detector (model L-2400 UV).

The olive leaf extract was resuspended in 10 mg/mL methanol, filtered through membrane with 0.45-μm pores (Fig. S1A), and analyzed by electrospray ionization-mass spectrometry (ESI-MS, Fig. S1B).

The phenolic compounds in Ex and Olv were evaluated using a UV/vis spectrophotometer (model SP-220; Biospectro, Curitiba, Brazil). Aliquots of the sample solution (50 μL) were mixed with 250 μL of Folin-Ciocalteau reagent (Blainski, Lopes & De Mello, 2013) and 750 μL of 20% sodium carbonate and the absorbance at 735 nm was measured after 2 h. A calibration curve was constructed using caffeic acid as the standard compound, and the results are expressed in terms of milligrams of caffeic acid/liter of sample.

The total phenol content of Olv was higher than that of Ex. The fatty acid content of Olv was analyzed by Shimadzu gas chromatography, according to the Hartman & Lago method (Mello & Pinheiro, 2012; Hartman & Lago, 1973), in which a 400-mg aliquot of the sample was converted to methyl esters using an ammonium chloride solution and sulfuric acid in methanol as an esterifying agent. The different types of fatty acids were identified by chromatography through a comparison of retention times of the samples and standards. Quantification was performed using area normalization, and the results were expressed as percentage per 100 grams of sample. The results are presented in Table S1.

Rats received 100 mg/kg of lyophilized Ex, administered by gavage daily throughout the experimental period. Using a pipette, 100 μL of Olv was carefully dispensed by dripping directly into the mouth of rats. Sepsis was induced by LPS treatment after 24 and 48 h and rats were subsequently treated with Ex, Olv, and Ex + Olv (24 and 48 h).

### Treatment protocol: omega-3 fish oil

Omega-3 fish oil was directly extracted from 1,000-mg capsules containing 21.3% EPA and 11.4% DHA. Using a pipette, 100 μL of Omg was carefully dispensed by dripping directly into the mouth of rats. Sepsis was stimulated by LPS treatment after 24 and 48 h and rats were subsequently treated with Omg (24 and 48 h).

### Treatment protocol: palm oil

The composition of the Pal used in this study was as follows: 42% saturated fatty acids (39–44% palmitic acid); 5–5.8% stearic acid; 43% monounsaturated fatty acids (38–46% oleic acid); 10–14% linoleic acid; 500–700 ppm carotenoids; and 600–1,200 ppm tocopherols, especially tocotrienols. Using a pipette, 100 μL of Pal was carefully dispensed by dripping directly into the mouth of rats. Sepsis was stimulated by LPS treatment after 24 and 48 h and rats were subsequently treated with Pal (24 and 48 h).

### Experimental groups

The experimental protocol was approved by the Ethics Committee of the Universidade Federal de São Paulo (CEP 0350/12) and was performed in accordance with the Brazilian guidelines for scientific animal care and use (Concea (CNDCDEA/MDCTEI), 2013; Brazilian Ministry of Science, Technology, Innovation and Communication, 2016).

Thirty male Wistar rats (weight: 230–250 g), not subjected to sepsis, were divided into five treated groups; Ex, Olv, Ex + Olv (ExOlv), Omg, and Pal. The baseline analysis values for these groups are presented in Table S2.

A total of 49 male Wistar rats (weight: 230–250 g) were divided into seven groups (*n* = 7). The CTL group was not submitted to sepsis. The LPS group was submitted to sepsis. Other rats were subjected to sepsis and were treated subsequently with Ex (LPSEx group), Ex + Olv (LPSExOlv group), Olv (LPSOlv group), Omg (LPSOmg group), and Pal (LPSPal group).

Rats were allowed free access to tap water and food during the experimental period. At 0 and 17 h, blood samples were collected from the orbital sinus and rats were maintained in metabolic cages for 24 h for urine collection. In some experiments, the urine was collected after 24 h and the volume was measured. Rats were euthanized, 48 h after the beginning of the experimental protocol, by intraperitoneal injection of a toxic dose of ketamine (90 mg/kg)/xylazine (10 mg/kg; Agribands de Brasil Ltda, Sao Paulo, Brazil). The right and left kidneys were then removed for immunohistochemical analysis. Biochemical parameters were measured in plasma and urine samples.

### Survival

A total of 62 male Wistar rats (weight: 230–250 g), were used exclusively to Survival experiment, and were divided into the following groups: CTL (*n* = 6), LPS (*n* = 16), LPSEx (*n* = 10), LPSExOlv (*n* = 8), LPSOlv (*n* = 7), LPSOmg (*n* = 8), and LPSPal (*n* = 7). Survival was assessed at 0, 24, and 48 h. Results were expressed as the percentage of surviving animals (%).

### Measurement of systolic blood pressure

Systolic blood pressure (SBP) was indirectly measured by tail plethysmography. Rats were placed in a warm chamber for 10 min and the cuff and wrist receiver were attached to the tail. Blood pressure was recorded using an electric sphygmomanometer coupled to a two-channel gould model 2200 S polygraph (Record 2200 S; Gould Inc., Cleveland, OH, USA). Measurements were taken at 0, 24, and 48 h, and the results are expressed as means ± SD.

### Creatinine clearance

Serum and urine creatinine levels were assayed spectrophotometrically at 0 and 48 h, according to standard procedures, using commercially available diagnostic kits (Labtest Diagnostica, Minas Gerais, Brazil). Creatinine clearance (mL/min) was calculated according to the following formula: (urine creatinine concentration × urine volume)/(serum creatinine concentration × 1,440).

### Lipidic peroxidation

To assess lipid peroxidation, levels of the peroxidation product, malondialdehyde, were determined by measuring thiobarbituric acid-reactive substances (TBARS). In this assay, the reactive substances in the test sample combine with thiobarbituric acid to form a red compound (Beuge & Aust, 1978). Urine samples were added to a solution of 0.375% thiobarbituric acid, 15% trichloroacetic acid, and 0.25 N HCl (Sigma, St. Louis, MO, USA). Subsequently, the samples were continuously agitated while heating to 95 °C for 20 min and were then allowed to cool to room temperature. Finally, the absorbance of the solution at 534 nm was determined using a spectrophotometer. Assays were performed at 0, 24, and 48 h. Results are expressed as nmol/mg creatinine.

### Serum cytokines

Tumor necrosis factor alpha (TNF-α), interleukin-1 alpha (IL-1α), interleukin-1 beta (IL-1β), interleukin-6 (IL-6), interleukin-10 (IL-10), and granulocyte-macrophage colony-stimulating factor receptor (GM-CSF), were measured in rat serum using a Luminex High Performance Assay (RnD Systems, Minneapolis, MN, USA), according to the manufacturer’s instructions. Assays were performed at 0, 17, and 48 h, and results are expressed as pg/mL.

### Immunohistochemistry

Hematoxylin and eosin staining was performed on kidney tissue sections. Paraffin-embedded tissues were cut into four-μm-thick sections on a rotary microtome (Leica Microsystems, Herlev, Denmark). The kidney slices were then deparaffinized, rehydrated, and boiled in a target retrieval solution (one mmol/L Tris, pH 9.0, with 0.5 mM EGTA) for 10 min to facilitate antigen binding. Nonspecific binding was prevented by incubating sections in PBS containing 1% BSA, 0.05% saponin, and 0.2% gelatin. Endogenous peroxidase activity was blocked by incubation in 5% H_2_O_2_ in absolute methanol for 10 min at 18–21 °C. Sections were then incubated overnight at 4 °C with primary antibodies against proliferating cell nuclear antigen (PCNA; diluted 1:500) and cleaved caspase-3 (diluted 1:100). Sections were washed and incubated with appropriate horseradish peroxidase-conjugated secondary antibodies (Dako, Glostrup, Denmark) for 1 h at room temperature. The sites of antibody-antigen reactions were visualized by staining with 0.5% 3,3′-diaminobenzidine tetrachloride (Dako) in 0.1% H_2_O_2_. The areas of PCNA were quantified using image analysis software (Leica DFC 310 FX, LAS software, version 3.8) to calculate the mean intensity value and expressed as a stained area. A total of 36 randomized microscope fields from different groups for cleaved caspase-3 were counted, with the same-sized square and the mean calculated and expressed as μm^2^.

### Statistical analysis

Results are expressed as the mean ± standard deviation (SD). Data were analyzed by Kaplan–Meier (survival curves), two-way analysis of variance (treated groups vs CTL and LPS groups), or simple linear regression analysis (treated groups versus Omg group), followed by a Bonferroni post hoc test. Values of *p* < 0.05 were considered statistically significant.

## Results

### Blood pressure

**Figure 1:**
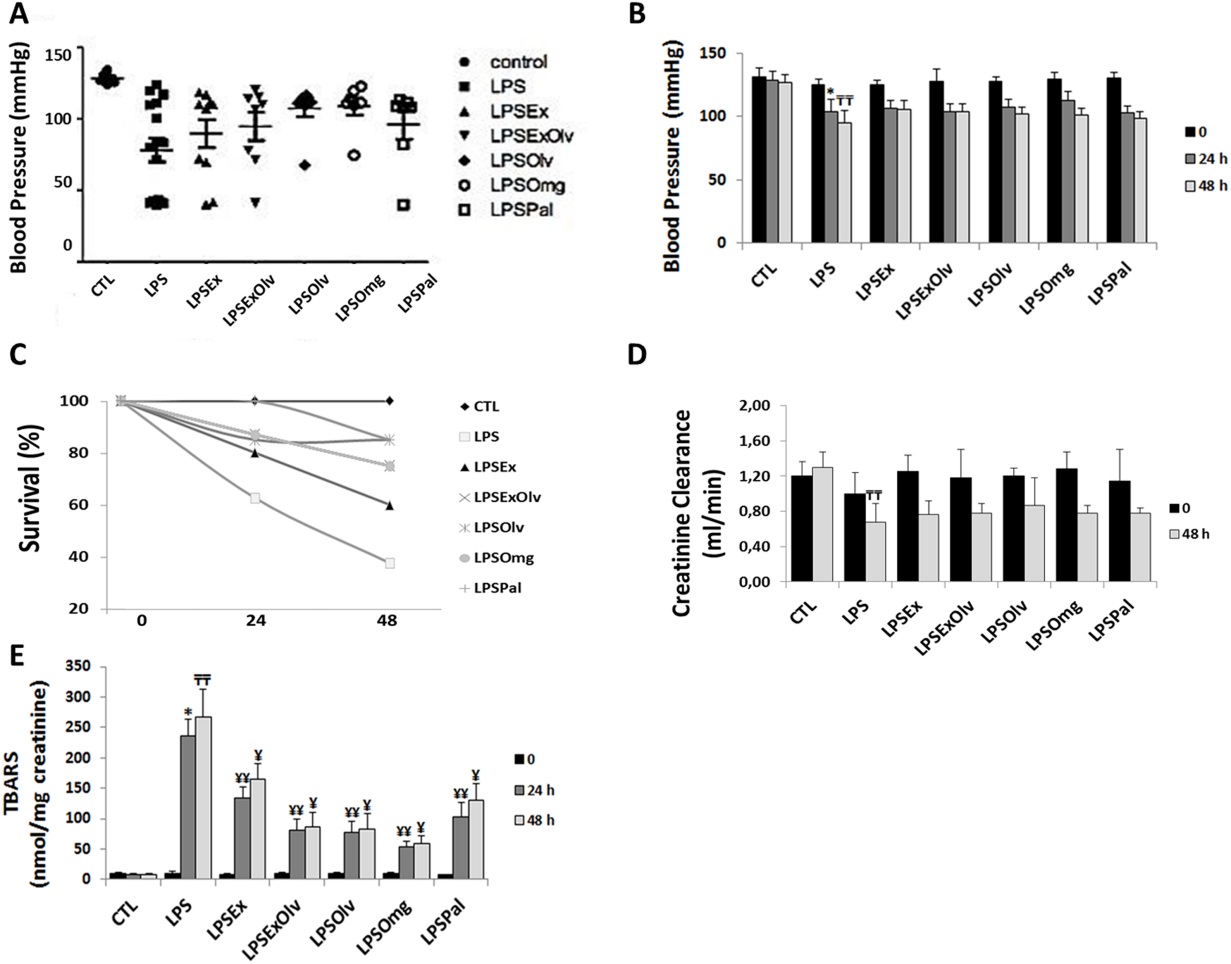
Physiological parameters analyses. (A, B) Systolic blood pressure. (C) Survival. (D) Creatinine clearance. (E) Quantification of thiobarbituric acid reactive substances (TBARS), in rats not subjected to sepsis, which were either untreated (CTL), rats subjected to sepsis (LPS), septic rats treated with an ethanolic extract of Brazilian olive leaf (LPSEx), Brazilian olive oil (LPSOlv), ethanolic extract of Brazilian olive leaf + Brazilian olive oil (LPSExOlv), palm oil (LPSPal), or omega-3 fish oil (LPSOmg). Data are reported as means ± SD. The significance level for the null hypothesis was set at 5% (*p* < 0.05). (*) LPS group at 24 h compared to the CTL group at 24 h, (╤╤) LPS group at 48 h compared to the CTL group at 48 h, (¥¥) for all groups at 24 h compared to the LPS group at 24 h, (¥) for all groups at 48 h compared to the LPS group at 48 h (ANOVA analysis followed by Bonferroni post hoc test).

Figure 1 shows the results of SBP measurements. Mean SBP values (Fig. 1A and 1B) of the LPS group were significantly lower than those of the CTL group after 24 h. Sepsis was characterized by hypotension. Treatments had no significant effect on blood pressure in septic rats. The blood pressure values for all groups are presented in Table S3.

### Survival

There were no deaths in animals kept under CTL conditions. A total of 37% (6 out of 16) of rats in the LPS group survived for the entire 48-h experimental period. In septic rats treated with Ex, the overall survival rate was 60% (6 out of 10). The survival rate was 75% (6 out of 8) in the ExOlv and Omg groups and 85% (6 out of 7) in the Olv and Pal groups. All treatments tested resulted in a significant increase in the mean survival rate of septic rats at 48 h in comparison to the LPS group (Fig. 1C). The survival data for all groups are presented in Table S3.

### Creatinine clearance

As shown in Fig. 1D, we observed a significant decrease in creatinine clearance in LPS group at 48 h, when compared to the CTL group, the sepsis-induced AKI. All treatments had no significant effect on creatinine clearance in septic rats. The values for all groups are presented in Table S3.

### Urinary levels of TBARS

Abnormal production of ROS may result in cellular injury through peroxidation of membrane lipids, protein denaturation, and DNA damage (Srivastava & Kumar, 2015). Figure 1E shows the urinary levels of TBARS in experimental rats and the antioxidant effect of experimental treatments. There was an increase in lipid peroxidation in LPS group, compared to the CTL group at 24 h. Conversely, the levels of TBARS were lower in all treated groups than in the LPS group, within their respective experimental periods, but Ex and Pal showed the lowest antioxidant effects. The lipid peroxidation data for all groups are presented in Table S3.

### Anti-inflammatory and proinflammatory mediators

**Figure 2:**
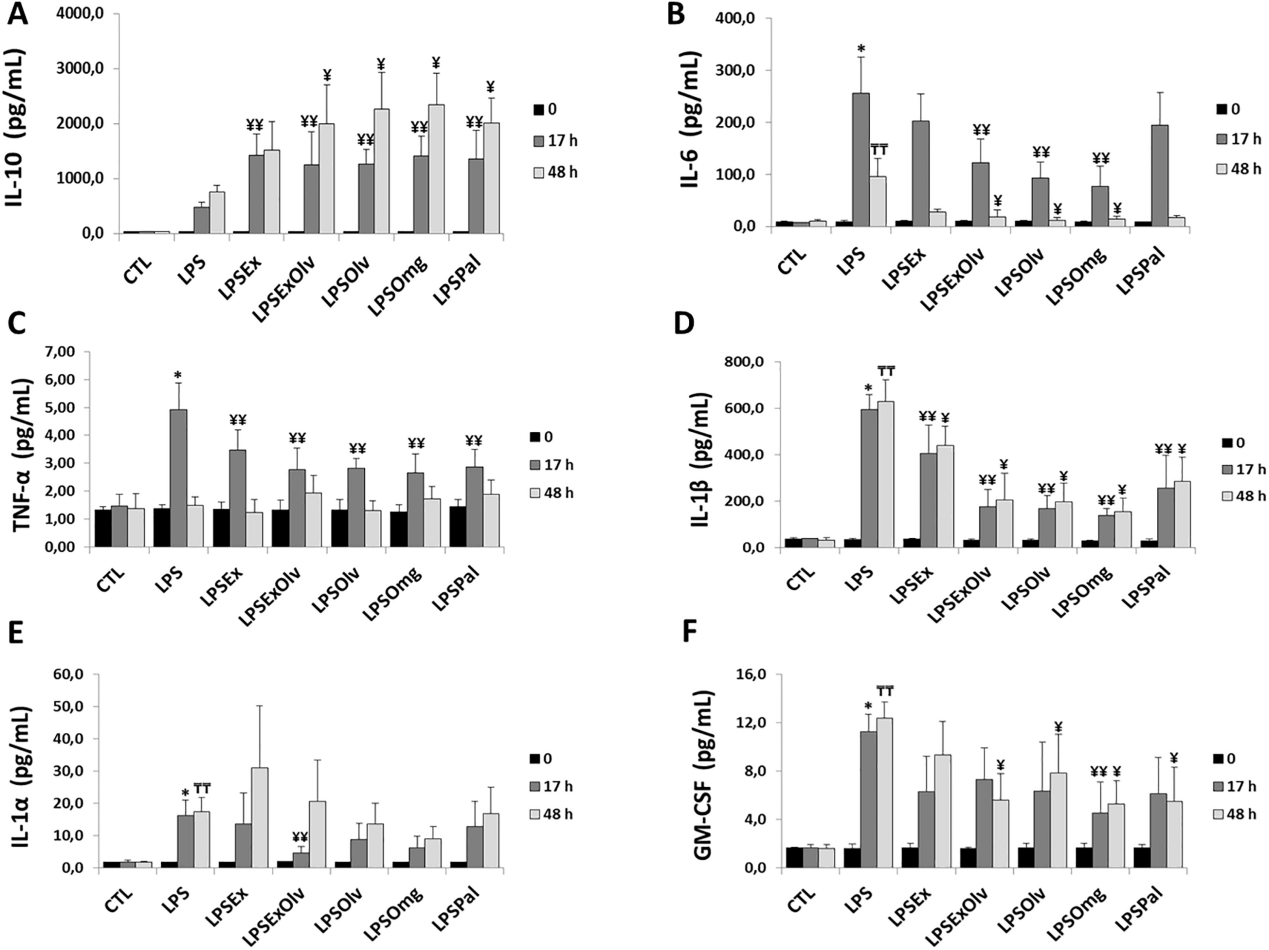
Dosage of anti-inflammatory and proinflammatory mediators. (A) Interleukin 10 (IL-10). (B) Interleukin 6 (IL-6). (C) Tumor necrosis factor alpha (TNF-α). (D) Interleukin 1β (IL-1β). (E) Interleukin 1α (IL-1α). (F) Granulocyte-macrophage colony-stimulating factor receptor (GM-CSF) in rats not subjected to sepsis, which were either untreated (CTL), subjected to sepsis (LPS), septic rats treated with ethanolic extract of Brazilian olive leaf (LPSEx), Brazilian olive oil (LPSOlv), ethanolic extract of Brazilian olive leaf + Brazilian olive oil (LPSExOlv), palm oil (LPSPal), or omega-3 fish oil (LPSOmg). Data are reported as means ± SD. The significance level for the null hypothesis was set at 5% (*p* < 0.05). (*) LPS group at 17 h compared to the CTL group at 17 h, (╤╤) LPS group at 48 h compared to the CTL group at 48 h, (¥¥) for all groups at 17 h compared to the LPS group at 17 h, (¥) for all groups at 48 h compared to the LPS group at 48 h (ANOVA analysis followed by Bonferroni post hoc test).

Interleukin-10 is an important anti-inflammatory cytokine (Latorre et al., 2014). IL-10 levels in the LPS group were not significantly different from those in the CTL group. When compared to the LPS group, all other groups showed higher levels of IL-10, within their respective experimental periods (Fig. 2A). The IL-10 levels for all groups are presented in Table S3.

Interleukin-6 is a reliable marker for the early diagnosis and follow-up of sepsis (Gabay, Lamacchia & Palmer, 2010). IL-6 levels increased in the LPS group at 17 and 48 h, compared to the CTL group. However, when compared to the LPS group, the LPSExOlv, LPSOlv, and LPSOmg groups showed significantly lower IL-6 levels at 17 and 48 h. IL-6 levels in the LPSEx and LPSPal groups were not significantly different from IL-6 levels in the LPS group, within their respective experimental periods (Fig. 2B). The IL-6 levels for all groups are presented in Table S3.

The secretion of proinflammatory cytokines, such as TNF-α and IL-1 (including IL-1α and IL-1β), in sepsis has been demonstrated in numerous studies (Cannon et al., 1990; Hesse et al., 1988). TNF-α levels increased in the LPS group when compared to the CTL group at 17 h. However, when compared to the LPS group, there was a significant decrease in TNF-α levels in all other treatment groups at 17 h. No significant differences in TNF-α levels were observed between experimental groups at 48 h, indicating a short half-life of TNF-α (Fig. 2C). The TNF-α levels for all groups are presented in Table S3.

The inhibition of IL-1β protects against LPS-induced sepsis (Kang et al., 2004). We observed higher levels of IL-1β in the LPS group after 17 h when compared to the CTL group. Inversely, IL-1β levels were lower at 17 and 48 h in other treatment groups than in the LPS group (Fig. 2D), suggesting anti-inflammatory effects of the tested oils and extracts.

Compared to the CTL group, IL-1α levels increased at 17 and 48 h in the LPS group, which is suggestive of systemic inflammation (Fig. 2E). There was no significant effect of extract or oil treatment on IL-1α levels, with the exception of the LPSExOlv group, in which a significant decrease was seen at 17 h, compared to the LPS group. The IL-1α levels for all groups are presented in Table S3.

Once released, TNF-α and IL-1 act on different target cells to promote the proliferation, activation, differentiation, and survival of macrophages (Witsell & Schook, 1992; Fahlman et al., 1994; Conte et al., 2006) and all these effects enhance proinflammatory responses during sepsis, we confirmed an increase in GM-CSF levels at 17 and 48 h in the LPS group, when compared to the CTL group. GM-CSF levels only decreased at 17 h in the LPSOmg group, but were not statistically different from the LPS group at 17 h in the other treatment groups. However, at 48 h, GM-CSF levels were lower in all treatment groups, except the LPSEx group (Fig. 2F). The GM-CSF levels for all groups are presented in Table S3.

### Cell proliferation and apoptosis in the kidney

**Figure 3:**
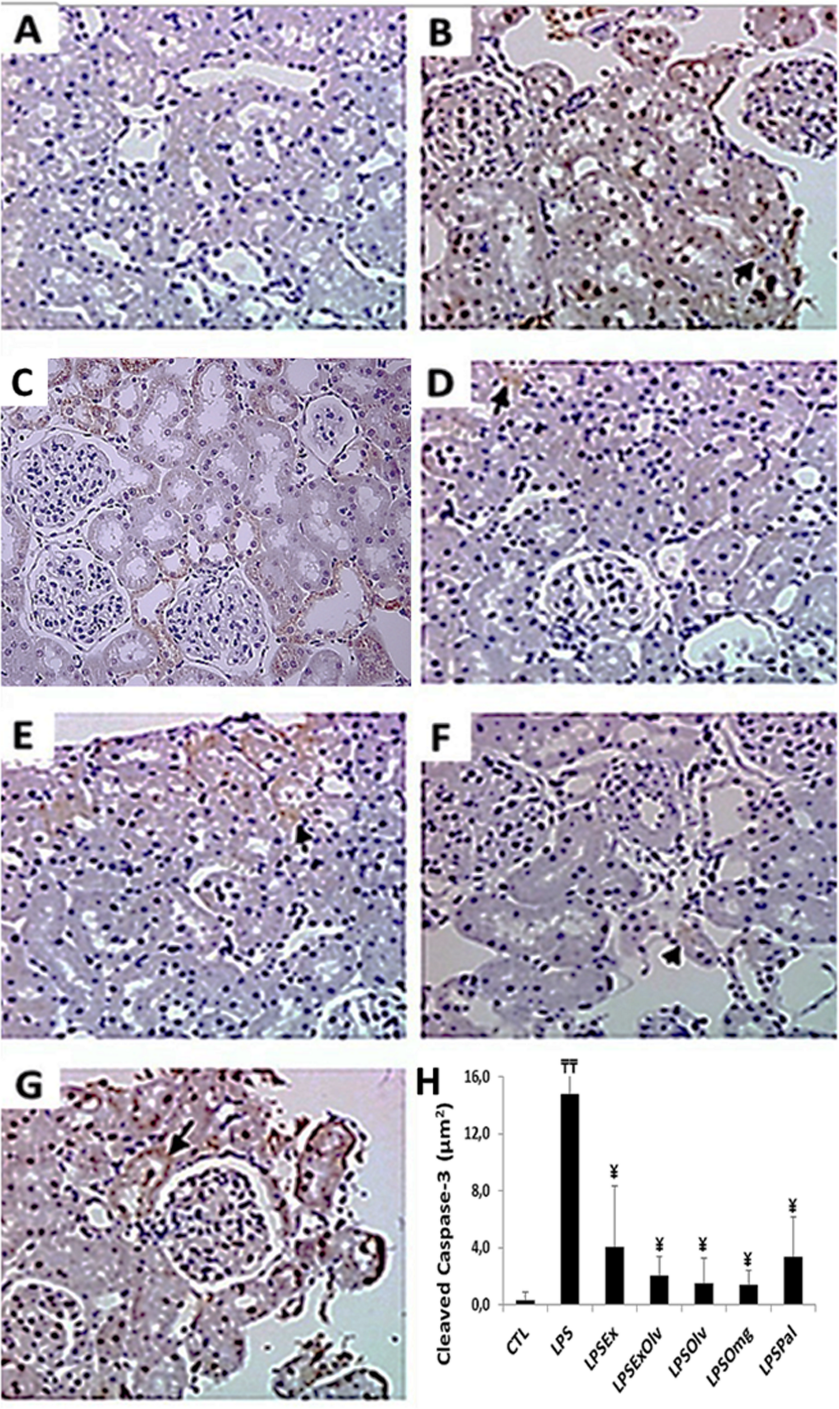
Light microscopy of kidney sections immunestained with cleaved caspase-3 (staining is shown in brown). (A) Rats not subjected to sepsis which were not treated (CTL). (B) Rats subjected to sepsis (LPS). (C) Septic rats treated with an ethanolic extract of Brazilian olive leaf (LPSEx). (D) Septic rats treated with Brazilian olive oil (LPSOlv). (E) Septic rats treated with ethanolic extract of Brazilian olive + Brazilian olive oil (LPSExOlv). (F) Septic rats treated with omega-3 fish oil (LPSOmg). (G) Septic rats treated with palm oil (LPSPal). (H) Quantitative analyses of kidney sections stained for cleaved caspase-3. Data are reported as means ± SD. The significance level for the null hypothesis was set at 5% (*p* < 0.05). (╤╤) LPS group at 48 h compared to the CTL group at 48 h, (¥) for all groups at 48 h compared to the LPS group at 48 h (ANOVA analysis followed by Bonferroni post hoc test).

The number of apoptotic cells per field was also analyzed by immunohistochemical detection of cleaved caspase-3 (Fig. 3). We observed an increase in apoptosis at 48 h in the LPS group when compared to the CTL group. The number of apoptotic cells in the LPSEx, LPSExOlv, LPSOlv, LPSOmg, and LPSPal groups was significantly lower the number of apoptotic cells in the LPS group (Fig. 3). The cleaved caspase-3 immunohistochemistry data for all groups are presented in Table S3.

**Figure 4:**
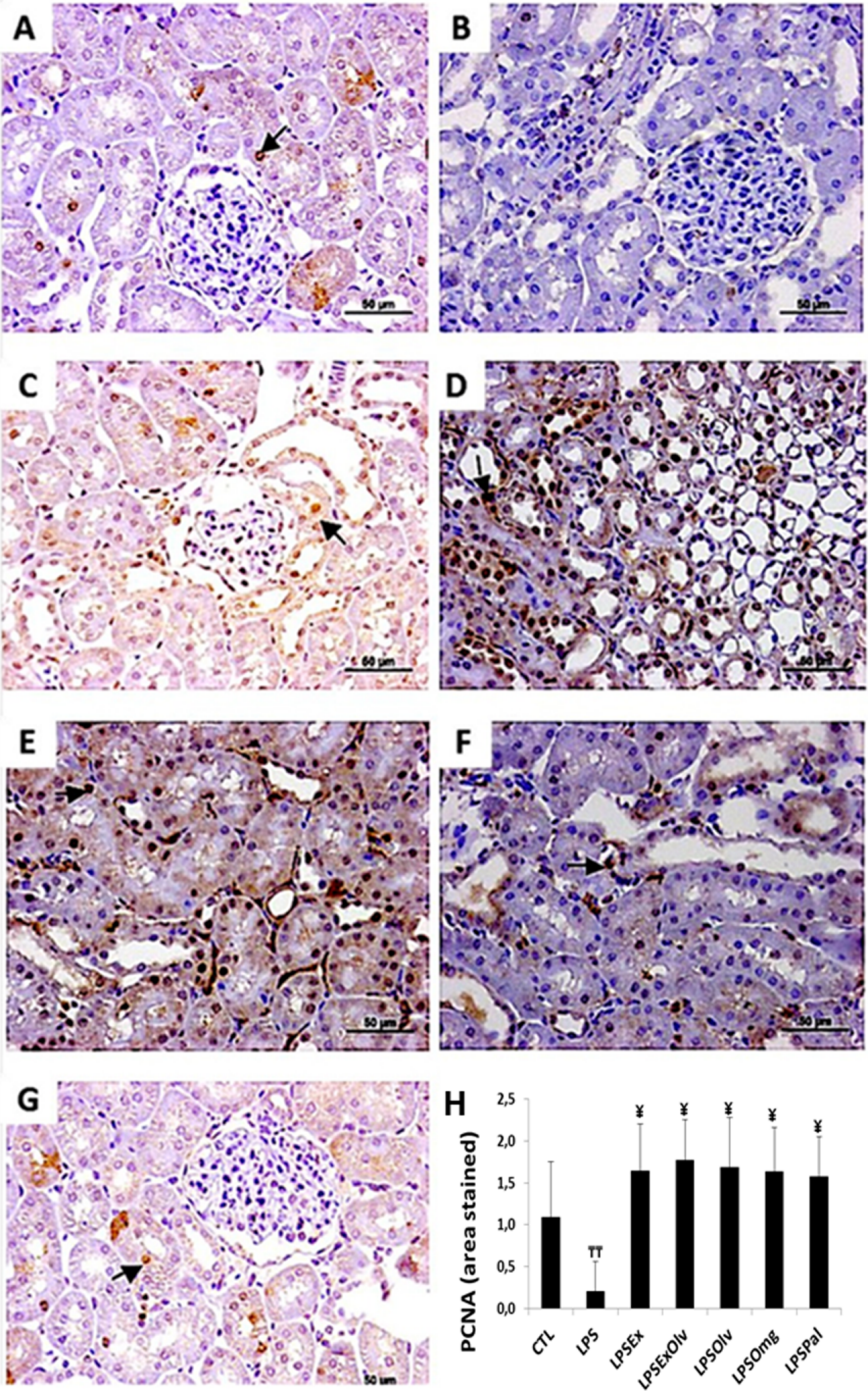
Light microscopy of kidney sections immunostained with PCNA (staining is shown in brown). (A) Rats not subjected to sepsis which were not treated (CTL). (B) Rats subjected to sepsis (LPS). (C) Septic rats treated with an ethanolic extract of Brazilian olive leaf (LPSEx). (D) Septic rats treated with Brazilian olive oil (LPSOlv). (E) Septic rats treated with ethanolic extract of Brazilian olive + Brazilian olive oil (LPSExOlv). (F) Septic rats treated with omega-3 fish oil (LPSOmg). (G) Septic rats treated with palm oil (LPSPal). (H) Quantitative analyses of kidney sections stained for cleaved caspase-3. Data are reported as means ± SD. The significance level for the null hypothesis was set at 5% (*p* < 0.05). (╤╤) LPS group at 48 h compared to the CTL group at 48 h, (¥) for all groups at 48 h compared to the LPS group at 48 h (ANOVA analysis followed by Bonferroni post hoc test).

Light microscopic images of kidney tissue sections are presented in Fig. 4. We observed a decrease in PCNA expression in the LPS group compared to the CTL group. However, a high level of PCNA expression was detected at 48 h in kidneys of the LPSEx, LPSExOlv, LPSOlv, LPSOmg, and LPSPal groups, when compared to the LPS group, indicating an increase in cell proliferation. The PCNA expression data for all groups are presented in Table S3.

### Comparative effects of olive and palm products and omega-3 fish oil

Table 1 compares the results of LPSEx, LPSOlv, LPSExOlv, and LPSPal treatments with the results of LPSOmg treatment. In our experimental conditions, only Olv showed similar results to Omg, since there was no significant difference in lipid peroxidation, IL-1β, and IL-6 data between these two treatments.

**Table 1:** Comparative effects of olive and palm products and omega-3 fish oil.

	Time (h)	LPSOmg	LPSOlv	LPSExOlv	LPSEx	LPSPal
**TBARS**	0	10.3 ± 1.7	9.90 ± 1.7	9.7 ± 2.2	7.3 ± 3.5	8 ± 1.9
	24	53.2 ± 10.3	76.5 ± 20	80.7 ± 19	134.3 ± 18.4*	102.6 ± 24.7*
	48	58.5 ± 13.2	82.3 ± 26.3	86.8 ± 23.5	164.9 ± 26.4**	130.1 ± 28.3**
**IL-6**	0	8.69 ± 1.0	9.99 ± 1.04	10.0 ± 1.5	10.1 ± 1.8	9.1 ± 1.4
	17	76.3 ± 39.4	92.7 ± 31.5	121.8 ± 45.8	202 ± 51.8*	194.2 ± 62.4*
	48	14.2 ± 4.7	12.1 ± 4.5	17.9 ± 13.3**	27.2 ± 5.5**	17.2 ± 3.9**
**IL-1β**	0	29 ± 3	32 ± 4	32 ± 5	37 ± 2	29 ± 8
	17	139 ± 29	166 ± 57	174 ± 77	404 ± 125*	256 ± 140*
	48	154 ± 59	198 ± 80	205 ± 114	439 ± 82**	285 ± 103**

**Notes:**

Quantification of thiobarbituric acid reactive substances (TBARS), interleukin 6 (IL-6) levels, and interleukin 1β (IL-1β) levels in septic rats treated with an ethanolic extract of Brazilian olive leaf (LPSEx), Brazilian olive oil (LPSOlv), ethanolic extract of Brazilian olive leaf + Brazilian olive oil (LPSExOlv), palm oil (LPSPal), and omega-3 fish oil (LPSOmg). Data are reported as means ± SD. The significance level for the null hypothesis was set at 5% (*p* < 0.05).

* For all groups at 17 or 24 h compared to the LPSOmg group at 17 or 24 h.

** For all groups at 48 h compared to the LPSOmg group at 48 h (simple linear regression analysis followed by Bonferroni post hoc test).

## Discussion

Systemic inflammatory response syndrome in septic patients is characterized by an exacerbation of inflammation, with increased levels of pro-inflammatory cytokines in the bloodstream. This stimulates an intense cellular response, characterized by the release of secondary mediators and the activation of granulocytes, which are responsible for the reactivation of phagocytic cells, which increases their oxygen consumption, causing the production of ROS and thus, forming a vicious cycle of inflammation (Pang et al., 1994; Noworyta-Sokołowska, Górska & Gołembiowska, 2013; Wiersinga et al., 2014).

Renal injury (Park et al., 2012; Stewart et al., 2005) and sepsis are associated with a significant increase of ROS and lipid peroxidation. In our experimental model, sepsis was characterized by hypotension and increase of IL-6, early marker of sepsis. Additionally, sepsis induced AKI was characterized by reduction of creatinine clearance.

The treatments tested in this study resulted in a decrease in lipid peroxidation in the urine of septic rats. This reduction may be related to the lower level of granulocyte activation observed under these experimental conditions.

Reactive oxygen species also mediates cell cycle arrest by activating inhibitory proteins. Furthermore, high concentrations of ROS have been shown to induce apoptosis and necrosis (Dong-Yun et al., 2003). PCNA is an auxiliary protein of DNA polymerase and it plays a fundamental role in the initiation of cell proliferation. Its expression is used as an index of renal regeneration (Park et al., 1997; Plotnikov et al., 2018). Therefore, we were interested in evaluating these parameters in the kidneys of rats in this study.

A decrease in the levels of cleaved caspase-3, an important marker of apoptosis, accompanied by an increase in PCNA expression, was observed in the kidneys of septic rats treated with all test substance, suggesting a mechanism of renal repair. However, we did not observe a normalization of creatinine clearance or blood pressure, which may need a longer treatment time to take effect.

When evaluating the hyperinflammatory state in septic rats, those all treatments showed a decrease in TNF-α and IL-1β levels, while those treated with Olv and Omg also showed a decrease in IL-6 levels. These results demonstrated important anti-inflammatory effects of all treatments tested, but especially for Olv and Omg.

Interleukin-10 is the main cytokine responsible for the regulation of the innate immune response (Kosaka et al., 2016). It is considered an anti-inflammatory cytokine that suppresses the production of proinflammatory cytokines (Barsig et al., 1995). All-treated septic rats showed an increase in IL-10 levels, indicating the induction of an anti-inflammatory response.

According to previous reports, the dysregulated expression of the cytokines, IL-6, TNF-α, and IL-1β, is associated with mortality in patients with sepsis (Stevens et al., 2017; Walley et al., 1996). A meta-analysis showed that TNF-α-targeted therapies result in only a 2% improvement in mortality compared to placebo (Lv et al., 2014). Similarly, IL-1 receptor agonist administration has shown limited clinical success (Opal et al., 1997). However, therapeutic interventions targeting individual cytokines have not conferred a significant clinical benefit.

Therefore, it is possible that the complications that develop in experimental sepsis and perhaps also in human sepsis, may be attenuated by therapeutic interventions that either reduce the levels of proinflammatory mediators or that restore the impaired adaptive and innate immune responses.

Among all therapeutic strategies, immunomodulation through the use of Omg has received significant attention and its effects are well established in experimental models of sepsis (Lu et al., 2017; Chen et al., 2018; Svahn et al., 2016; Dehkordi et al., 2015; Frimmel et al., 2016). In view of this, we compared the effects of all test substances used in this study with the effects of Omg.

Treatment Ex and Pal were beneficial in septic rats in this study. These treatments increased survival rate and did not aggravate inflammation, but they were not as effective as Olv and Omg. The reduced protection against sepsis observed after Ex treatment may be explained by the significantly lower total phenol content in this extract when compared to Olv (Mello & Pinheiro, 2012). The phenol content in several plants is related to its antioxidant properties (Piluzza & Bullitta, 2011). Additionally, the reduced protection observed after Pal treatment can be explained by the high percentage of palmitic acid (Fattore & Fanelli, 2013). Previous studies showed that palmitic acid-induced inflammatory injury in many tissues and organs as myocardial (Wang et al., 2017), brain (Ortiz-Rodriguez et al., 2019), liver (Xu et al., 2016), and kidney (Liu et al., 2018).

Interestingly, only Olv and Omg decreased the sepsis-induced secretion of the inflammatory cytokines, TNF-α, IL-6, and IL-1β and stimulated the secretion of the anti-inflammatory cytokine, IL-10. Olv and Omg increased survival rate and down-regulated ROS levels in septic rats, which was accompanied by a lower level of granulocyte/macrophage activation. Although Olv and Omg were not effective at improving renal function or blood pressure during the first 24 to 48 h of sepsis, they did induce a higher level of cell proliferation in the kidney, which was accompanied by a decrease in apoptosis, suggesting a mechanism of renal repair.

These results support the hypothesis that Brazilian olive oil (Olv) has similar effects to Omg on LPS-induced sepsis in rats. A population-based cohort study showed that adherence to a Mediterranean-style diet is related to lower risk of sepsis (Gray et al., 2018). This diet is based on vegetables, fruits, legumes, cereal, and omega 3 fatty acid rich fishes. According to our results, we can speculate that olive oil rich diet could also contribute to the lower the risk of sepsis. Nevertheless, other studies are necessary to better address this question.

## Conclusion

The administration of Brazilian olive oil and Omg had considerable beneficial effects on LPS-induced sepsis, mainly due to reversal of the hyperinflammatory state. Therefore, these two natural food substances have similar effects and must be studied further in order to provide a better understanding of the pathophysiological mechanisms underlying sepsis and thus, enable the development of more effective therapeutic interventions.

## Supplemental Information

10.7717/peerj.7219/supp-1 Supplemental Information 1 Evaluation of olive leaf extracts. **Figure S1A.** Olive leaf extracts were analyzed at Phytobios/Centroflora Brazil, using a high-performance liquid chromatography system, and analyzed by electrospray ionization-mass spectrometry **(Fig. S1B).**

10.7717/peerj.7219/supp-2 Supplemental Information 2 Evaluation of olive oil. **Table S1.** The fatty acid content of Olv was analyzed by Shimadzu gas chromatography. The results were expressed as percentage per 100 grams of sample.

10.7717/peerj.7219/supp-3 Supplemental Information 3 The baseline analysis values of rats not subjected to sepsis and treated. **Table S2**. Data are reported as means ± standard deviation for systolic blood pressure (SBP), creatinine clearance (CrCl), thiobarbituric acid reactive substances (TBARS), tumor necrosis factor alpha (TNF-α), interleukin 6 (IL-6), interleukin1α (IL-1α), interleukin 1β (IL-1β), interleukin 10 (IL-10), granulocyte-macrophage colony-stimulating factor receptor (GM-CSF) and number of surviving animals in rats not subjected to sepsis and treated with an ethanolic extract of Brazilian olive leaf (Ex), Brazilian olive oil (Olv), ethanolic extract of Brazilian olive leaf + Brazilian olive oil (ExOlv), palm oil (Pal), or omega-3 fish oil (Omg).

10.7717/peerj.7219/supp-4 Supplemental Information 4 The analysis values of rats not subjected to sepsis, which were either untreated (CTL), rats subjected to sepsis (LPS), or septic rats treated. **Table S3.** Data are reported as means ± standard deviation for systolic blood pressure (SBP), creatinine clearance (CrCl), thiobarbituric acid reactive substances (TBARS), tumor necrosis factor alpha (TNF-α), interleukin 6 (IL-6), interleukin1α (IL-1α), interleukin 1β (IL-1β), interleukin (IL-10), and granulocyte-macrophage colony-stimulating factor receptor (GM-CSF), in rats not subjected to sepsis, which were either untreated (CTL), rats subjected to sepsis (LPS), septic rats treated with ethanolic extract of Brazilian olive leaf (LPSEx), Brazilian olive oil (LPSOlv), ethanolic extract of Brazilian olive leaf + Brazilian olive oil (LPSExOlv), palm oil (LPSPal), or omega-3 fish oil (LPSOmg).

10.7717/peerj.7219/supp-5 Supplemental Information 5 Raw data exported from the results, used for data analysis and figure preparation.

## Additional Information and Declarations

### Data Availability

The following information was supplied regarding data availability: The raw numeric data is available as a Supplemental File.
