# Supplementary figures and images for: Comparison of olive leaf, olive oil, palm oil, and omega-3 oil in acute kidney injury induced by sepsis in rats
Source: PeerJ. 2019 Jul 9;7:e7219. doi: 10.7717/peerj.7219 (PMC6625600; doi:10.7717/peerj.7219)

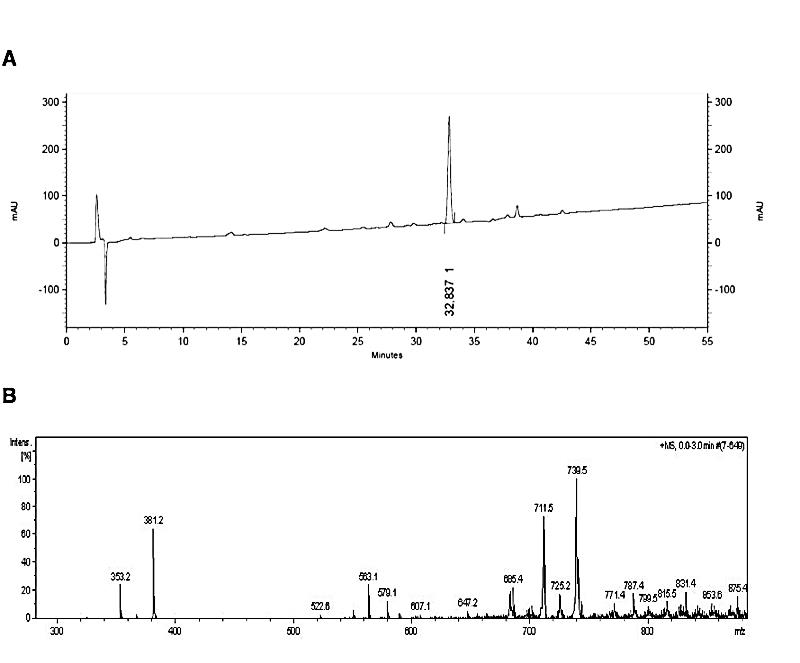

Supplement: Supplemental Information 1 — Figure S1A. Olive leaf extracts were analyzed at Phytobios/Centroflora Brazil, using a high-performance liquid chromatography system, and analyzed by electrospray ionization-mass spectrometry (Fig. S1B). [file peerj-07-7219-s001.png]
