# Supplementary material for: Comparison of olive leaf, olive oil, palm oil, and omega-3 oil in acute kidney injury induced by sepsis in rats
Source: PeerJ. 2019 Jul 9;7:e7219. doi: 10.7717/peerj.7219 (PMC6625600; doi:10.7717/peerj.7219)
Supplement: Supplemental Information 2 — Table S1. The fatty acid content of Olv was analyzed by Shimadzu gas chromatography. The results were expressed as percentage per 100 grams of sample. [file peerj-07-7219-s002.docx]

| **Fatty acids** |  | **Olive oil** |
| --- | --- | --- |
| **Saturated** | C 16:0 palmitic | 18 % |
|  | C 17:0 margaric | 0,1 % |
|  | C 18 estearic | 1,63 % |
|  | C 20:0 araquidic | 0,4 % |
|  | C 22:0 behenic | 0,1 % |
| **monoinsaturated** | C 16:1 ω 7palmitoleic | 2,29 % |
|  | C 17:1 cis-10-heptadecenoic | 0,19 % |
|  | C 18:1 ω 9oleic | 59,9 % |
|  | C 20:1 ω 11cis-11-eicosenoic | 0,3 % |
| **polyunsaturated** | C 18:2 ω 6 linoleic | 11,8 % |
|  | C 18:3 ω 3α-linolenic | 0,76 % |
