# Supplementary material for: Comparison of olive leaf, olive oil, palm oil, and omega-3 oil in acute kidney injury induced by sepsis in rats
Source: PeerJ. 2019 Jul 9;7:e7219. doi: 10.7717/peerj.7219 (PMC6625600; doi:10.7717/peerj.7219)
Supplement: Supplemental Information 3 — Table S2. Data are reported as means ± standard deviation for systolic blood pressure (SBP), creatinine clearance (CrCl), thiobarbituric acid reactive substances (TBARS), tumor necrosis factor alpha (TNF-α), interleukin 6 (IL-6), interleukin1α (IL-1α), interleukin 1β (IL-1β), interleukin 10 (IL-10), granulocyte-macrophage colony-stimulating factor receptor (GM-CSF) and number of surviving animals in rats not subjected to sepsis and treated with an ethanolic extract of Brazilian olive leaf (Ex), Brazilian olive oil (Olv), ethanolic extract of Brazilian olive leaf + Brazilian olive oil (ExOlv), palm oil (Pal), or omega-3 fish oil (Omg). [file peerj-07-7219-s003.docx]

|  | **Time (h)** | **Ex** | **ExOlv** | **Olv** | **Omg** | **Pal** |
| --- | --- | --- | --- | --- | --- | --- |
| **SBP** | **0** | 126.7 ± 6.9 | 124.7 ± 7.9 | 122.0 ± 8.5 | 124.0 ± 9.4 | 125.7 ± 7.3 |
|  | **24 h** | 123.8 ± 8.1 | 119.8 ± 12.2 | 124.5 ± 5.0 | 122.7 ± 6.2 | 123.8 ± 6.9 |
|  | **48 h** | 127.2 ± 5.2 | 121.0 ± 7.9 | 120.5 ± 12.2 | 124.3 ± 7.0 | 126.5 ± 7.0 |
| **CrCl** | **0** | 1.25 ± 0.20 | 1.18 ± 0.30 | 1.20 ± 0.10 | 1.28 ± 0.20 | 1.14 ± 0.10 |
|  | **48 h** | 1.00 ± 0.20 | 1.14 ± 0.14 | 1.20 ± 0.08 | 1.00 ± 0.10 | 1.15 ± 0.30 |
| **TBARS** | **0** | 8.37 ± 0.97 | 7.45 ± 0.86 | 7.45 ± 0.87 | 7.42 ± 0.86 | 9.23 ± 1.07 |
|  | **24 h** | 7.63 ± 0.89 | 6.72 ± 0.78 | 8.13 ± 0.95 | 9.08 ± 1.05 | 7.83 ± 0.91 |
|  | **48 h** | 8.77 ± 1.03 | 9.13 ± 1.07 | 9.18 ± 1.09 | 8.95 ± 1.05 | 8.40 ± 0.97 |
| **TNF-α** | **0** | 1.32 ± 0.05 | 1.38 ± 0.05 | 1.38 ± 0.06 | 1.38 ± 0.16 | 1.27 ± 0.12 |
|  | **17 h** | 1.37 ± 0.12 | 1.31 ± 0.03 | 1.31 ± 0.21 | 1.33 ± 0.13 | 1.65 ± 0.10 |
|  | **48 h** | 1.45 ± 0.18 | 1.11 ± 0.22 | 1.16 ± 0.06 | 1.36 ± 0.1 | 1.49 ± 0.14 |
| **IL-6** | **0** | 8.27 ± 0.40 | 8.67 ± 0.4 | 9.10 ± 0.56 | 8.23 ± 1.05 | 7.77 ± 0.55 |
|  | **17 h** | 7.70 ± 1.51 | 7.84 ± 1.91 | 9.33 ± 0.55 | 8.84 ± 1.01 | 10.10 ± 0.26 |
|  | **48 h** | 9.58 ± 1.68 | 7.60 ± 2.23 | 7.64 ± 1.42 | 8.25 ± 201 | 10.13 ± 1.31 |
| **IL-1 α** | **0** | 1.67 ± 0.42 | 1.83 ± 0.46 | 1.70 ± 0.69 | 1.97 ± 0.35 | 1.82 ± 0.44 |
|  | **17 h** | 1.82 ± 0.48 | 1.70 ± 0.53 | 1.82 ± 0.45 | 1.81 ± 0.45 | 2.16 ± 0.24 |
|  | **48 h** | 1.73 ± 0.57 | 1.53 ± 0.42 | 1.68 ± 0.30 | 1.77 ± 0.62 | 2.02 ± 0.07 |
| **IL-1β** | **0** | 34.67 ± 3.06 | 31.50 ± 3.60 | 33.13 ± 0.81 | 32.87 ± 3.06 | 34.1 ± 3.48 |
|  | **17 h** | 37.33 ± 1.53 | 29.47 ± 2.55 | 30.33 ± 4.04 | 31.60 ± 2.62 | 37.33 ± 6.43 |
|  | **48 h** | 31.77 ± 4.97 | 28.67 ± 7.57 | 28.67 ± 5.03 | 35.67 ± 6.03 | 38.67 ± 2.52 |
| **IL-10** | **0** | 31.45 ± 2.25 | 33.67 ± 1.15 | 31.36 ± 1.96 | 34.23 ± 6.81 | 35.63 ± 2.90 |
|  | **17 h** | 34.67 ± 2.89 | 34.19 ± 4.88 | 32.66 ± 4.71 | 36.3 ± 10.51 | 33.35 ± 5.89 |
|  | **48 h** | 30.82 ± 2.55 | 30.80±4.02 | 33.81 ± 2.7 | 34.72 ± 5.49 | 35.31 ± 2.86 |
| **GM-CSF** | **0** | 1.60 ± 0.35 | 1.66±0.33 | 1.59 ± 0.01 | 1.64 ± 0.06 | 2.11 ± 0.70 |
|  | **17 h** | 1.64 ± 0.36 | 1.63±0.36 | 1.60 ± 0.17 | 2.01 ± 0.34 | 1.57 ± 0.09 |
|  | **48 h** | 1.57 ± 0.10 | 1.64±0.27 | 1.56 ± 0.01 | 2.07 ± 0.48 | 2.13 ± 0.24 |
| **Survival** | **0** | 6 | 6 | 6 | 6 | 6 |
|  | **17 h** | 6 | 6 | 6 | 6 | 6 |
|  | **48 h** | 6 | 6 | 6 | 6 | 6 |
